# Annual Meeting of Constituents

**Published:** 1919-12-27

**Authors:** 


### METROPOLITAN HOSPITAL SUNDAY FUND.

### Annual Meeting of Constituents.

The annual meeting of constituents of this Fund was held at the Mansion House on Thursday, December 18, the Right Hon. the Lord Mayor presiding. There was a good attendance.

The **Secretary** (Mr. Arnold James) announced that letters regretting the absence of the writers had been received from the Bishop of Islington, the Rev. A. A. Harland, Sir Heath Robinson, Sir Henry Burdett, the Rev. A. W. Parsons, the Right Hon. Lord Leith of Fyvie, and the Rev. A. H. Stone.

The **Chairman** reminded the gathering of the conditions governing the conduct at the annual meetings by reading Law VI. He then moved that the annual report be taken as read.

**Mr. Robert Holland-Martin, C.B.** (Hon. Treasurer), seconded, and it was agreed to.

**Mr. Holland-Martin** then proposed that the report of the Council for 1919 be received and adopted. He said there were one or two matters to which, as Hon. Treasurer, he wished to draw attention. The first was the very great loss which the Fund had sustained in the past year by the death of his predecessor in this office, Sir Ernest Tritton. Sir Ernest's loss was very great, because he knew how to work with great effect and to encourage others to work. And, though this Fund was only one of his many activities, it was one which he worked almost his hardest for.

The total receipts this year were £86,232, the second largest collection in the history of the Fund, the high-water mark having been achieved in 1918, when more than £92,000 was realised. In this latter figure, however, there was included £5,000 from the American Red Cross. This year the donations were £11,271, and the Church collections £40,307, a slight falling-off compared with last year, when there was £43,000 from that source. But these collections had been somewhat difficult. The general uncertainty of the outlook had been several times represented to him, and particularly the future of hospitals, whether the Ministry of Health was going to take a large part in their management, and whether the State would contribute to their upkeep. These questions had made some people a little shy about giving. It was hoped to do a good deal more during the coming year by house-to-house collections. The churches in connection with which this was done in the past year were indicated by a star, and it would be seen that in almost every case the result was very satisfactory. Help was forthcoming last year, too, from the County of London branch of the British Red Cross Society, who collected for the Fund £500. He found them very ready to help, and he hoped that next year, by increased enthusiasm being induced, the receipts from this source would be considerably augmented. In 1919 the amount received by the Fund from its great benefactor's (Mr. George Herring) estate was £21,667—a very large amount. (Applause.)

**The Ven. the Archdeacon of London** seconded, and desired to associate himself with all that had been said about the late Sir Ernest Tritton. At the same time the Fund was to be congratulated on the appointment of Mr. Holland-Martin to succeed Sir Ernest.

The motion was carried unanimously.

**The Rev. Dr. Kilgour** (St. Columba, Pont Street) proposed the following resolution:

That the following members of Council for 1919—the Bishop of Rochester, the Bishop of Southwark (R.C.), the Archdeacon of London, Canon Curtis, the Rev. J. Monro Gibson, the Rev. J. G. Curry, the Rev. Simpson Johnson, Lord George F. Hamilton, Lord Burnham, the Hon. Sir W. H. Goschen, Sir James Reid, Bart., Sir Heath Harrison, Bart., R. Ernest Alexander, Esq., Edward Dent, Esq., John M. Morgan, Esq., John Robarts, Esq.—be and are hereby re-elected for 1920, with the addition of the Rev. Dr. Carter, St. Dionis', Fulham; the Rev. Prebendary Gough, M.A., Vicar of Brompton; the Rev. H. G. Monroe, M.A., Vicar of Wimbledon; the Rev. G. N. Walsh, M.A., Vicar of Hammersmith; the Rev. G. C. Wilton, M.A., St. Anne's, Soho; Sir Francis Green, Bart.; Sir Alfred Tritton, Bart.; R. V. Somers-Smith, Esq., to fill vacancies.

**Metropolitan Hospital Sunday Fund**—(*continued*).

He said the large sum which his Church, the Church of Scotland, "St. Columba," had been able to collect had been the result not of any large contribution, but, following persistent and earnest pulpit references, a great number of small contributions on Hospital Sunday. This effort was one of the greatest pieces of good work done in London; but he thought it ought to be recognised in future, in connection with this Fund, that unless contributions were increased somewhat in the same ratio as were those at St. Columba last year, the money realised would not equal in spending power that previously received. He commended the resolution to the meeting.

**Rev. Thomas Jackson** (Brunswick Hall, Whitechapel) seconded the motion, and in doing so expressed the hope that the sum of £100,000 would be raised this year. It was merely a question of willing to do it. He hailed from the aristocratic neighbourhood of Whitechapel, where the Mission and the Institute drew 80 per cent. of their constituency from the Brady street area. Two years ago he told his people that when they were ill they could go to the hospital, because they could not afford to have a doctor, and it was thus incumbent upon them to help the hospitals. He told them they must raise £50, and they did it. This year he asked them to commemorate peace by raising £200, and they did it. (Applause.) If the Bishop of London had been present to-day he would almost have blushed to hear that the poor men of Whitechapel sent £45 more to this Fund than did the worshippers at St. Paul's Cathedral. (Applause.) He believed that thousands of pounds additional could be got from the poor if only the effort were carried out in the right way. He asked one poor woman to subscribe half-a-crown a year. She said very few half-crowns came her way, but she would willingly give a penny a week. This he accepted as a concession! He instanced the case of two women who went to a certain hospital a few weeks ago. They had been instructed to be in attendance at one o'clock, and arrived at twenty minutes to one, but were not attended to until six o'clock. It would be readily realised what a wait of five hours meant to delicate people. It was merely a matter of better arrangement on the part of the hospital authorities, in order to obviate what, he was sure, was an unintentional hardship to the poor.

The resolution was carried unanimously.

**Sir William Church** in proposing that the next Hospital Sunday be fixed for June 27, 1920, said it was necessary that the date should be fixed as early as possible because charitable institutions arranging entertainments within a limited period of Hospital Sunday were penalized on the grant received for the year, so that there should be no such things as hospital bazaars or similar fêtes in the interests of any hospital that would be likely to clash with Hospital Sunday or occur within a short time of the date fixed.

**Sir Dyce Duckworth** seconded the proposition, and it was unanimously agreed to.

**Rev. W. Hardy-Harwood** proposed that Law IX. be amended by the deletion of the word "Vice-Chairman," and the substitution of "Vice-President" therefor. It had been discovered that the same office was described in one part of the Constitution as "Vice-President," and in another as "Vice-Chairman," and the letter of the resolution was merely the correction of that error. The spirit of it, however, was the desire to give the most important person after the President his proper position, and they were therefore very anxious that the Vice-President, who would act in the absence of the Lord Mayor (whom they were very fortunate in always having as President), should have full recognition.

**Sir John Bell** seconded the resolution, and it was carried unanimously.

**Mr. R. Holland-Martin** proposed that a very hearty vote of thanks be given to the Lord Mayor for presiding, especially in view of the fact that he had had a very busy day owing to a great event—the visit of the Prince of Wales to the City.

The vote of thanks having been heartily accorded,

The **Chairman**, in returning thanks, said that he was very glad to be able to aid in the work. Hospital Saturday and Hospital Sunday had been of immense help to the hospitals. He felt their thanks were due to Mr. Arnold James, the Secretary, and his assistant for the work they had done during the year. It was with pleasure that he publicly thanked them, and he was sure all would agree that those thanks were deserved. (Applause.)

